# Allergic diseases in infancy II–oral tolerance and its failure^☆^

**DOI:** 10.1016/j.waojou.2021.100586

**Published:** 2021-11-19

**Authors:** Mathias Hornef, Oliver Pabst, Isabella Annesi-Maesano, Manja Fleddermann, Erika von Mutius, Monika Schaubeck, Alessandro Fiocchi

**Affiliations:** a Institute of Medical Microbiology, RWTH Aachen University Hospital, Pauwelsstr. 30, Aachen, 52074, Germany; b Institute of Molecular Medicine, RWTH Aachen University, Pauwelsstr. 30, Aachen, 52074, Germany; c Epidemiology of Allergic and Respiratory Diseases Department, IPLESP, French Institute of Health and Medical Research and Sorbonne University, Medical School Saint Antoine, 27 Rue Chaligny, Paris, 75012, France; d HiPP GmbH & Co. Vertrieb KG, Georg-Hipp-Straße 7, Pfaffenhofen, 85276, Germany; e Dr. von Hauner Children's Hospital, University of Munich, Lindwurmstr. 4, Munich, 80337, Germany; f Division of Allergy, Pediatric Hospital Bambino Gesú (IRCCS), Piazza di Sant’Onofrio 4, Rome, 00165, Italy

**Keywords:** Allergy, Allergy prevention, Oral tolerance, Window of opportunity

## Abstract

**Objective:**

The early window of opportunity describes the timeframe after birth in which essential interactions of the immune system and the newly developing microbiota take place. The infant's immune system has to be reactive to invading pathogens and at the same time tolerant to dietary antigens. If the mechanisms of defense and tolerance induction are disturbed, the risk of infections or allergies is increased.

**Method:**

This is a narrative review of the recently published information on the topic of neonatal intestinal development and mechanisms of oral tolerance and summarizes the discussions and conclusions from the 8^th^ Human Milk Workshop.

**Results:**

The early postnatal period sets the stage for life-long host-microbiome interaction. In this early phase, specific developmental mechanisms ensure physiologic interaction with the developing microbiota. Innate and adaptive immune cells interact in a concerted way to induce and uphold oral tolerance. Factors in human milk can support this induction of tolerance and simultaneously protect against infection and allergy development.

**Conclusion:**

Understanding the developmental mechanisms in this early phase of immune system development is the first step to develop strategies of pathology prevention. As human milk protects the infant from infections, and aids to develop a tolerogenic immune response, further knowledge on the protective factors in human milk and their effect on the immune system is required.

## Introduction

Under physiological conditions, the ingestion of food antigens can lead to local and systemic immune non-responsiveness — ie,. oral tolerance. Immune mechanisms of oral tolerance induction seem to be connected with mechanisms of tolerance to commensal microbes. Upon birth, a baby transits from the sterile and protected intrauterine milieu towards an environment full of microbial and dietary stimuli. Starting on the first day of life, neonates must be able to ward off pathogens and simultaneously tolerate commensal microorganism or food antigens.

In this context, knowledge of the mechanisms of a balanced host-microbe interaction during the neonatal period and during infancy is important for understanding the reciprocal mechanisms of a physiological host-food interaction.

The kind and intensity of the host-microbial interaction at mucosal surfaces might promote or protect from disease development in later life. Epidemiological observations show that exposure to a diverse microbial environment or a high incidence of infections seems to protect against allergic or autoimmune diseases.^1^^,^^2^ Limited early exposure to such microbial environments and the subsequently altered immune response are referred to as the "hygiene hypothesis". The fact that early infancy might be the crucial time window for exposure to specific microbial stimuli is regarded as the window of opportunity.^2^

However, which microbial stimuli provide protection has not yet been resolved. Recent studies have started to specify the mechanisms behind the results of several observational studies, identifying specific components of the intestinal microbiota that offer protection. Vatanen et al were able to demonstrate that geographical differences in the prevalence of autoimmune diseases are linked to varying abundance of *Bacteroides* and *E. coli* in the infant enteric microbiota. The cell membrane lipopolysaccharides (LPS) of these 2 commensals is known to differ in its acylation pattern. This structural difference determines the potential to stimulate the LPS receptor, Toll-like receptor (TLR) 4. Differences in the innate immune priming could therefore influence immune homeostasis and contribute to the development of allergies in later life.^3^ These observations highlight the importance of the microbe-host interaction during this early time window, with a long-term influence on health throughout life.

Increasing numbers of studies confirm that alterations of the microbe-host interaction in this early time window — for example, through antibiotic therapy — have pronounced immunological effects and increase the risk to develop allergic symptoms in later life, as recently shown in a meta-analysis.^4^ This raises the question which developmental, anatomical, and immunological changes characterize and define this neonatal window?

## The early postnatal period sets the stage for life-long host-microbiome interaction

The postnatal establishment of the enteric microbiota exemplifies the particular situation of infants. The neonatal microbiota differs in 4 important aspects from the adult microbiota: bacterial density, bacterial diversity, overall bacterial composition, and compositional stability over time. While recent results clearly demonstrate the absence of a prenatal or placental microbiome, there is no doubt about microbial stimuli released in the maternal gut reaching the fetus.^5^ The microbiota establishes rapidly after birth. Microorganisms colonize the infant's body — for example, the skin and the gut during the first hours after birth. Approximately 48 hours after birth, bacterial density (ie, the number of bacteria per gram of feces) reaches adult levels.^6^ Other characteristics of the microbiota, on the other hand, develop later: bacterial richness — meaning the number of different bacterial species — will not reach adult levels until after the first 2 years of life in humans. Due to low bacterial richness, the infant's microbiota is also highly prone to variations and therefore shows low stability. This high susceptibility to compositional deviations results in a low resistance to colonization by pathogens. Consequently, the neonatal immune system needs to be equipped with age-specific mechanisms to instantly react to pathogen exposure, compensating for its lack of an already established adaptive immunity.

## Age-dependent mucosal differences

The neonatal immune system is often referred to as being “immature”, though recent data show that the neonatal immune response is rather “specific” and “unique”.^7^ Better insight into the mechanisms of this distinct immunity as well as the mucosal surface development is crucial for the identification of ways for nutritional or non-nutritional interventions.

Since it is unethical to obtain human tissue from healthy neonates, animal models are an important tool to unravel physiologic mechanisms of structural and functional alterations in the mucosal surfaces. Therefore, murine models are still required for analyzing complex interactions of environmental factors, microbes, and the host's epithelial and immune system development at the same time. However, developmental differences between mice and humans must be considered when results of murine models are translated to human conditions.

Differences in neonatal intestinal architecture and gene expression appear to be a strategy to meet age-specific challenges of the postnatal and weaning phase. By using murine models, age-dependent differences of the mucosal tissue were observed for (1) the cell composition and turn-over of the epithelial barrier and its gene expression profile, (2) antigen uptake, and (3) antigen recognition and presentation.

The intestinal epithelial barrier is made up of intestinal epithelial cells (IECs), forming a single-cell layer on the surface of the intestinal mucosa, which is securely sealed by tight-junction proteins. This epithelial barrier is important for the physical separation of the high number of microbes in the intestinal lumen from the underlying largely sterile tissue. Crypt-villus migration of IECs — the passage from the newly formed cells in the intestinal crypt to the sloughed cells at the villus tip — is reduced in neonates.^8^ The associated cell shedding and regeneration are important mechanisms in the prevention of pathogen invasion. Consequently, reduced cell shedding might enable pathogens to form IEC-adherent micro-colonies, which explains the susceptibility of neonatal mice to enteropathogenic *E. coli* (EPEC) infections, for example.^9^

Paneth Cells (PC) are located at the small intestinal crypt base in close proximity to the epithelial stem cell compartment. Since different antimicrobial peptides (AMPs) are secreted to enforce the spatial segregation of microbes and the IEC layer, PC are regarded as key players for barrier integrity, innate defense, and microbial community regulation.^10^ Expression of these PC-derived AMPs is reduced in neonatal mice (day 3) as compared to adult (day 21) mice.^8^ This contrasts sharply with the high number of intestinal microbes, as low AMP levels would leave the neonates susceptible to infections. Instead, the cathelicidin-related antimicrobial peptide (CRAMP) is produced by the intestinal epithelium during the neonatal phase. In mice, expression of CRAMP is not limited to crypt-based PC but found along the epithelial lining. Interestingly, CRAMP was also found on the skin and in the breast milk of both mice and humans; thus, it might play a particularly important role for early host-microbial interaction.^11^

Goblet cells contribute to the intestinal barrier by secreting different mucus glycoproteins that prevent the direct contact of luminal microbes with the intestinal lining. The mucus structure and quantity are different in the postnatal period, compared to adult mice: in the murine neonatal intestine, a reduced production of mucus is observed, as epithelial exposure to microbial structures varies in early age.^12^ The mucus layer sustains the separation of intestinal microbes from the epithelial lining. Furthermore, it is also an important site for microbe-host interaction, as certain commensals are capable of metabolizing mucins. Therefore, the host's mucus production and microbiota composition influence each other.^13^

Microfold cells (M cells) are the major route of antigen uptake in the adult intestine, as they are capable of forwarding luminal antigens to the Peyer's patch (PP)-resident immune cells for presentation to the adaptive immune system.^14^ The neonatal murine gut is devoid of fully matured M cells. This absence may contribute to the high susceptibility of young infants for systemic infections with non-typhoidal *Salmonella* infections.^12^ In adults, the probable infectious route of *Salmonella* is via M cell transcytosis, which leads to subsequent immunological control in the underlying PP. In case of entry via the IEC route, *Salmonella* would be warded off efficiently by constant cell migration and shedding.^15^ In neonates, however, both pathways are impaired, possibly contributing to the neonatal high susceptibility to *Salmonella* infections.^12^

The recognition of microbial patterns by IECs and immune cells alike underlies ontogenetic changes. The recognition of gram-negative bacterial LPS via toll-like receptor 4 (TLR4) mediates proinflammatory immune responses in adults. Neonates, however, display a transient postnatal tolerance toward TLR4 ligands.^16^ A similar phenomenon is also observed at systemic sites.^17^ This process might be crucial in facilitating postnatal establishment of host-microbial homeostasis. The mechanistic basis for this neonatal TLR-mediated epithelial tolerance is posttranscriptional down-regulation of the signaling molecule interleukin 1 receptor-associated kinase 1 (IRAK-1). The resulting transient unresponsiveness, which might contribute to an increased susceptibility to infections, is compensated by protective human milk components (eg, alarmins).^17^^,^^18^ TLR-mediated tolerance of IECs toward LPS was altered in mice delivered by cesarean section.^16^ This supports the hypothesis that the birth mode might influence the establishment of postnatal immune homeostasis beyond microbial exposure.

Another example of neonatal-specific microbial recognition is the developmentally regulated epithelial expression of certain TLRs, which vary in their abundance during the postnatal phase. TLR3 expression is low in neonates and increases during weaning. TLR3 recognizes double-stranded (ds) RNA (ribonucleic acid), and its low expression in neonates might explain the age-dependent susceptibility of infants toward infection with rotavirus, a known dsRNA virus.^19^ A better understanding of pathogen susceptibility mechanisms in neonates is important, as repeated intestinal infections are inversely related to the risk of allergic diseases later in life.^20^

In contrast to TLR3, expression of TLR5 is enhanced at the neonatal IEC lining and declines during weaning. TLR5 mediates recognition of flagellin — a protein within the flagellum (a lash-like filamentous appendage facilitating mobility of bacteria). High postnatal expression is IEC-specific, as immune cells do not show age-dependent TLR5 expression. Using murine competitive colonization experiments, Fulde et al were able to show that high neonatal TLR5-expression favored intestinal colonization of non-flagellated bacteria. They demonstrated how the host actively shapes its own early microbiota.^21^ This highlights the importance of epithelial-mediated mechanisms in supporting the establishment of intestinal microbial homeostasis.

Developmental changes are not limited to innate defense mechanisms but extend to adaptive immunity as well. Soon after birth, thymus-emigrant T cells (TC) colonize the murine intestine, although initially exclusively the PP.^22^ These TC remain naïve throughout the neonatal phase, despite the presence of nutritional and microbial antigens.^23^ Around weaning, the exposure of mucosal tissue to milk-derived epithelial growth factors (EGF) is reduced and a transient phase of TNF-α and IFN-γ driven immune stimulation ends the active suppression of TC.^24^ This process is also referred to as a weaning reaction, in which the induction of CD4^+^ regulatory TC in the lamina propria (LP) is started. These regulatory TC differ in their transcriptional profile and pattern of activation markers, compared to CD4^+^ TC in adults. Therefore, postnatal suppression of TC maturation might mitigate the preservation of a broad TC receptor repertoire. Especially microbiota-dependent, peripherally generated regulatory T cells (Tregs) play an important role in the initiation of tolerance and colonize the small intestine from weaning onwards.^22^^,^^25^ All these developmental changes illustrate how neonates cope with the sudden exposure to microbial and food antigens and how they facilitate the establishment of homeostasis in different phases.^26^ Moreover, soluble factors in human milk support the immune homeostasis in the postnatal period.^27^, ^28^, ^29^

The postnatal window of opportunity is a central developmental stage in the maturation of the infant's immune system. It includes numerous non-redundant, host-dependent, and environmental stimuli, which mature the infant's defense strategies in a concerted way in order to ensure physiological tolerance as well as efficient antimicrobial host defense. As these different phases might depend on each other, the concept of a layered immunity was introduced to describe the mechanism of microbe-host interaction, which is crucial to provide homeostatic mucosal defense and oral tolerance.^26^

## Mechanisms of oral tolerance induction in neonates

During the neonatal phase — which is accompanied by barrier establishment, microbiota engraftment and maturation of the immune system — an important immunologic mechanism must be established: tolerance toward food antigens. Ingestion of food antigens normally results in local and systemic immune unresponsiveness, called oral tolerance.^30^

Tolerance of food antigens does not stem automatically from ignorance — a lack of recognition or detection of antigens — but rather from a complex, concerted, and active tolerogenic response comprising various immune cell types and mechanisms.

It is essential for infants to develop a balanced immune response towards food antigens and commensals, while simultaneously defending against potential pathogens. Encounters with pathogens and commensals in the neonatal window of opportunity has long-lasting effects on the later response toward food antigens. If induction of these mechanisms is perturbed, tolerance toward foods might be lost. This indicates the need to identify factors modulating oral tolerance induction in earlier life and to define mechanisms and compounds facilitating oral tolerance induction. A mechanistic understanding of oral tolerance may eventually improve nutritional and non-nutritional strategies in allergy prevention.^30^

However, information on the complex pathways and cellular interactions of tolerance induction are still scarce. Presumably, multiple mechanisms and external factors are involved and consequently impaired tolerance and development of allergies seem complex and require a precise definition of the particular processes studied. Mechanisms of oral tolerance to food protein differ from important aspects of tolerance to the microbiota: Oral tolerance to food affects local and systemic immune responses, whereas tolerance to commensals does not mitigate systemic reactions. For both, however, the gut and gut-associated lymphoid tissue (GALT) seem to be the major site of immune induction, as will be discussed below.

## How does the immune system acquire tolerance to food antigen?

**Fig. 1:**
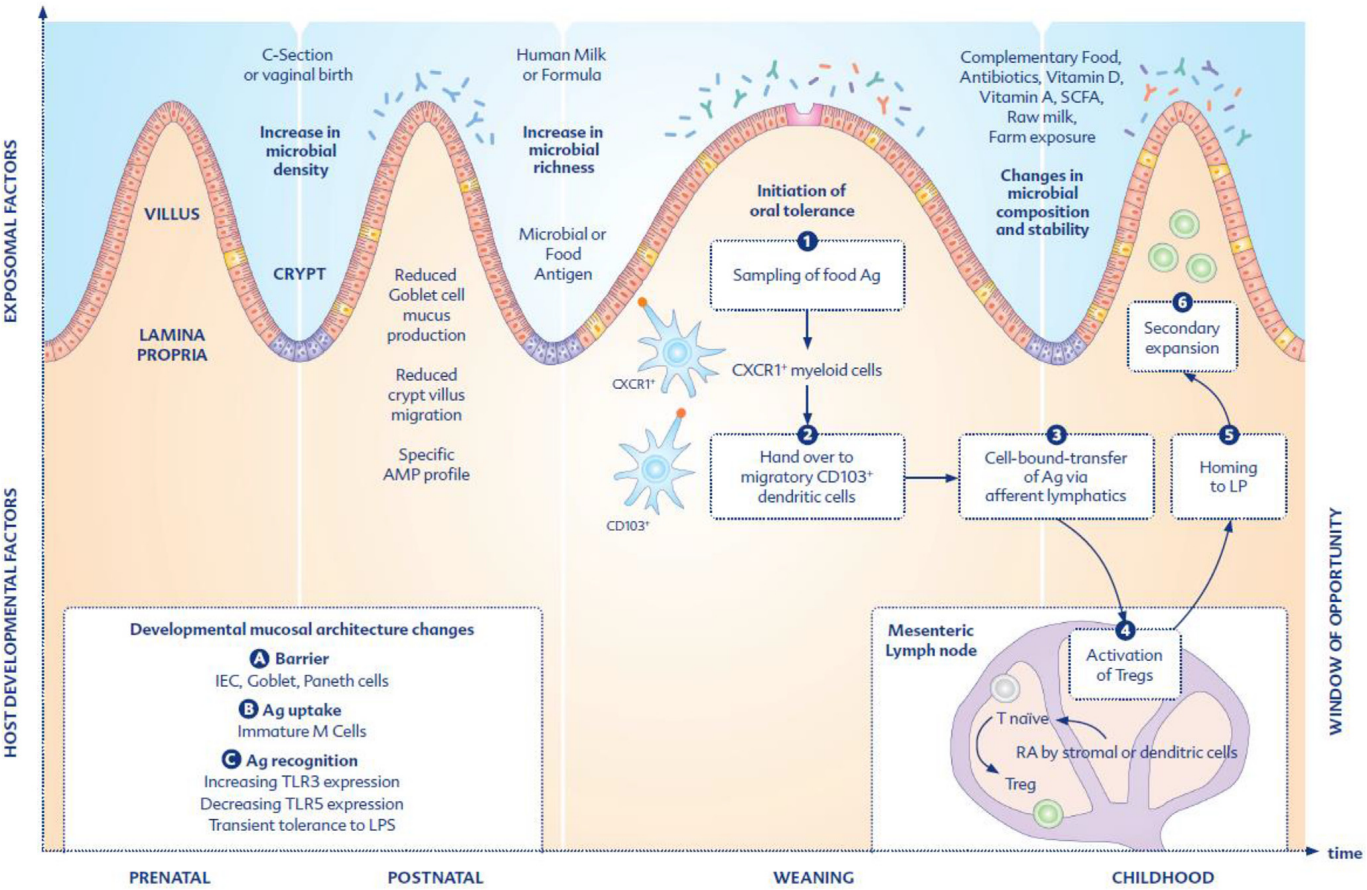
The window of opportunity — Development of the intestinal mucosal immune system and mechanisms of oral tolerance induction. During birth, a large number of microbes quickly colonizes the infant gut. The birth mode — vaginal birth or cesarean section (C-section) — influences the colonization by microbial species, which rapidly increases in density. The neonatal mucosa is initially devoid of crypt-residing Paneth cells (purple), the main producers of antimicrobial peptides (AMP). Thus, the postnatal and adult AMP profiles differ. The neonatal immaturity of the goblet cells induces a changed expression of mucins and a thinner mucus barrier. During the postnatal phase, microbial richness increases. During the milk-feeding period, *Lactobacilli*, *Streptococci* and *Bifidobacteria* dominate the intestinal microbiota. (1) Microbial as well as food antigens enter into the GALT or lamina propria via M cells and goblet cell associated channels (GAPs). In addition, the uptake via CXCR1+ myeloid cells, which collect luminal antigens by cellular protrusion extending through the gut epithelium into the intestinal lumen, is discussed. Regular transepithelial transport and paracellular leakage may enable antigen entry into the gut tissue. (2) The antigens are handed over to CD103+DCs co-expressing chemokine receptor 7 (CCR7). (3) Antigens are mostly transported in a cell-bound fashion by these CCR7+CD103+DCs to the mesenteric lymph nodes (mLN). (4) In the mLN, amongst others, TGF-β, IL-10 and retinoic acid (RA) promote the differentiation and activation of Tregs. (5) Consecutively, CCR9 mediated transfer of Tregs to the LP results in a (6) secondary expansion of Tregs. With weaning and the introduction of solid foods, the bacterial composition changes, and Bacteroidetes, for example, increase. Components in complementary food contribute to the changed bacterial composition by providing different amounts of dietary fibres, for example, which are metabolized by the intestinal microbiota.

The infant gut is exposed to an array of food proteins from human milk or — when breastfeeding is not possible, infant formula — and later complementary foods. In non-allergic infants, the immune system acquires an active tolerance towards food antigens. Like microbial antigens, food antigens are closely watched by the immune system. Antigen-presenting cells (APCs) — mostly dendritic cells (DCs) in the LP — will sample, process and present the antigens to adaptive immune cells. As tolerance towards food has long-term consequences, the adaptive immune system is pivotal in allergy prevention (Fig. 1).

At the time of TC differentiation, food antigens are not necessarily present in the thymus. Therefore, central tolerance — ie, the elimination of auto- or food-reactive lymphocytes during lymphocyte development cannot sufficiently explain oral tolerance. Instead, oral tolerance needs to be induced in the mature lymphocyte compartment.

In murine models, oral tolerance is usually induced by feeding a specific antigen, followed by immunization with the respective antigen.^31^ Combination of these models with TC receptor transgenic mouse strains indicated a key role of Tregs.^32^ Tregs play a central role in oral tolerance, as the transfer of Tregs into non-tolerant recipients is sufficient to transfer tolerance in a murine allergy-model.^33^ At least for the question of Treg mediated oral tolerance we have a framework model to answer key questions in food tolerance: Where is Treg expansion induced and where is it sustained? Which is the most important APC to induce Treg tolerance? What factors might contribute to tolerance development? At what point during the postnatal period or infancy is this interaction most successful?

## Where? – The gut associated lymphoid tissue as site of tolerance induction

The gut as an organ comprises the largest number of immune cells and is continuously exposed to a wide array of food proteins and commensals. Division of the immune response into inductive and effector sites facilitates efficient discrimination between harmful and innocuous antigens. Inductive sites include the gut-draining mesenteric lymph nodes (mLNs) and the organized structures of the GALT such as PP and isolated lymphoid follicles. In contrast, the effector sites comprise immune cells distributed throughout the intestinal epithelium and LP.^34^

A major route of intestinal antigen uptake is via M cells.^35^ Suzuki et al observed that oral tolerance is particularly effective when the antigens are directly targeted to M cells.^36^ However, tolerance can be induced in the absence of PP and M cells, and Kraus et al. showed that tolerance can be induced in gut loops devoid of PP as efficiently as in loops containing PP.^37^ We may therefore conclude that PP and M cells are not generally required for oral tolerance induction. In addition to the entry into the GALT, luminal antigens can enter directly into the intestinal LP. Multiple mechanisms have been suggested that support the sampling of antigens: 1) CXCR1^+^ myeloid cells collect luminal food antigens or microbial antigens by cellular protrusion extending through the gut epithelium into the intestinal lumen;^38^^,^^39^ 2) Antigens may enter the gut LP through goblet cell-associated antigen passage (GAPs) and tolerance to food antigens is impaired in the absence of GAPs;^40^ and 3) Regular transepithelial transport and paracellular leakage may enable antigen entry into the gut tissue. In all cases, antigens can be taken up by DCs located in the LP, which transport the antigens via afferent lymphatics toward the mLNs. In fact, mLNs are the central structure of oral tolerance anatomy, and seem to play a non-redundant function in oral tolerance induction.^41^ By using antigen-specific TC, Worbs et al were able to show that removal of mLNs prevented induction of oral tolerance.^42^ Antigens can reach the mLNs in free/unbound form, or via transport by migratory DCs (Figure). Especially the DC-mediated transport from the small intestine to the mLNs seems to be a crucial step in tolerance induction and dependent on chemokine receptor 7 (CCR7), as in CCR7-deficient mice no induction of oral tolerance was possible.^42^

When DC-bound antigens reach the mLNs, organ-specific anatomical structures, as well as non-hematopoietic stromal cells support oral tolerance induction. Stromal cells create the microanatomy of secondary lymphoid organs — spleen and lymph nodes— by defining distinct compartments and providing immunomodulatory signals.^43^ Stromal cells in mLNs and peripheral LNs differ in their composition and transcriptomic profile. This might put mLNs in a superior position for inducing TC homing to the gut.^44^^,^^45^

In summary, oral tolerance is initiated in the PP as well as LP and activated in the mLNs that drain the small intestine. After the activation of Tregs in the mLNs, homing of Tregs back to the LP is essential, as no permanent oral tolerance could be maintained in mice with impaired gut homing.^46^ Therefore, the LP seems to be the site of Treg expansion, supported by LP-specific CXCR1+ myeloid cells and locally produced IL-10.^46^^,^^47^

Free food antigens can also be detected in the serum of mice shortly after feeding. The portal vein transports blood from the intestine to the liver. When blood flow to the liver is prevented, no oral tolerance is induced.^48^^,^^49^ Similar to the mLNs, the liver also contains specific cells — such as liver sinusoidal endothelial cells, Kupffer cells, and conventional liver DCs, — which can induce tolerance rather than proinflammatory responses.^50^

In conclusion, the induction of oral tolerance is a complex multi-site and multi-step procedure including hematopoietic as well as non-hematopoietic cells.

## Who? – Induction of oral tolerance depends on the concerted action of innate and adaptive immune cells

DCs appear to be the most important APCs in oral tolerance: CXCR1+ myeloid cells sample luminal antigens from the lumen via extrusions through the intestinal barrier. However, these cells are non-migratory and only have low Treg-inducing capacity.^51^ Another subset of DCs, expressing the integrin chain CD103, is the major population of DCs carrying antigens from the intestine to the mLNs. CD103^+^ DCs are largely excluded from entering the bloodstream, suggesting that CD103^+^ DCs represent LP-derived migratory DCs.^52^ Cell-bound antigen transport from the LP to the mLNs by these migratory CD103^+^ DCs is important for oral tolerance induction.^53^ Free antigens, which are passively drained from the intestine, appear to remain immunologically inconspicuous and do not elicit T cell proliferation.^54^

CD103^+^ DCs are crucial for inducing Tregs, the central adaptive immune cells in oral tolerance. However, different subsets of IL-10 secreting Tregs contribute to oral tolerance induction. Of most interest here are FoxP3+ expressing Tregs. Foxp3 is the main transcription factor driving the differentiation and function of these important immune regulators. Also within the Foxp3+ Tregs, a further distinction can be made according to the site of induction. While natural Tregs (nTregs) are generated in the thymus, induced Foxp3+ Tregs (iTregs) are generated from naive T cells in the peripheral immune system. At least in murine models, iTregs seem to be essential in increasing oral tolerance.^46^^,^^55^ These iTregs are crucial for oral tolerance maintenance and involve lymphoid organs and mucosal sites alike.

## What? – Factors influencing oral tolerance development

The individual phases of the aforementioned immunological processes can be influenced by external factors such as diet and microbiota.

CD103+ DCs and stromal cells in the mLNs are particularly efficient to metabolize vitamin A to retinoic acid (RA). Vitamin A conversion is essential for CD103+ DCs to maintain their tolerance-inducing phenotype, including through activation, expansion and gut homing of Tregs.^56^^,^^57^ Sun et al were able to show that CD103+ DCs derived RA can act as a cofactor to TGF-β, thereby helping in the conversion of naive CD4^+^ TC to Tregs.^58^ RA alone was enough to induce the gut-homing molecules CCR9 and α4β7 integrin on TC *in vitro*.^57^ By administering vitamin A to vitamin A-deprived animals, oral tolerance development could be restored.^59^ Another study showed faster tolerance induction upon vitamin A supplementation in mice.^60^ However, these observations were made under pathological vitamin A deprivation. To our knowledge, no vitamin A-induced improvement of oral tolerance has yet been shown in humans.

Some studies suggest that vitamin D might augment intestinal Treg frequency, thereby promoting tolerance.^61^^,^^62^ This is why mechanisms and clinical effects of vitamin D on the immune system are extensively studied. Lower levels of vitamin D were found in infants with cow's milk allergy, accompanied by lower FoxP3 expression levels, which were predictive of slower acquisition of tolerance.^63^, ^64^, ^65^, ^66^

Besides direct effects of vitamins on the immune cells, indirect dietary-induced effects are possible as well. Diets are an important modulator of the intestinal microbiota. For example, the increased ingestion of fiber supports tolerogenic immune responses; a response that is likely mediated by short-chain fatty acids (SCFA).^67^ Microbe-derived butyric acid, was repeatedly shown to promote tolerance by increasing the number of activated Tregs and supporting innate tolerogenic mechanisms.^68^, ^69^, ^70^ In addition, the gut microbiota might also modify oral tolerance development directly, *i.e.* independent of its produced metabolites such as SCFA. TLRs recognize microbial signals and modulate Treg signaling.^71^ Certain components of the commensal microbiota are repeatedly observed to induce Tregs, an important mechanism explaining the tolerogenic potential of probiotic bacteria.^70^^,^^72^^,^^73^

## When? – The postnatal period as an important time window for oral tolerance induction

The first exposure to oral antigens occurs soon after birth, or even *in utero*.^60^ Therefore, mechanisms for oral tolerance induction start early in life. However, the exact timing is not known, as most mechanisms of oral tolerance induction were studied in adult mice. When using adult models, the unique physiology of neonates and the protecting factors provided by maternal milk (eg, TGF-β or immunoglobulins) are neglected. In addition, drawing a direct comparison between humans and murine models is difficult, due to the relative immaturity of the murine neonate gut and shorter lactation period. However, initial studies have provided an insight into the question of the relevant time window and the correlating cellular mechanisms which are critical for oral tolerance induction. In a study by Turfkruyer et al, neonatal mice were exposed to soluble ovalbumin (OVA) through maternal milk in either the first, second or third week of life.^61^ After weaning started (4 weeks of life), the mice were observed for several weeks. At 6–8 weeks of life, which corresponds to an almost adult mouse, their immune response to OVA was assessed. It was found that the administration of antigen at the end of the lactation period induced oral tolerance. In contrast, the allergic reaction was only partially prevented in mice receiving the antigen in their first week of life. This again underlines the often discussed importance of a strong epithelial barrier in allergy prevention. Additionally, while the numbers of CD103+ DCs were similar throughout their first weeks of life, their ability to metabolize vitamin A to RA was significantly lower during the neonatal phase and increased upon weaning. This indicates that reduced RA secretion in CD103+ DCs seems to be one causal factor for a limited oral tolerance during the neonatal phase.^60^ Whether supplementation of vitamin A in mothers or neonates can improve oral tolerance is still under debate, due to the disparity in results across human trials.^74^ Furthermore, no data about the timing of RA generation in human neonates is available. This clearly shows that further knowledge about cellular mechanisms throughout the window of opportunity is essential for future nutritional preventative strategies.

## Conclusion

The early postnatal period sets the stage for life-long host-microbiome interaction and oral tolerance induction. In this window of opportunity, the age dependent mucosal development and immune system interactions establish pathogen defense and food tolerance. Hereby the concerted interplay between innate and adaptive immune cells, the gut epithelial barrier and the developing microbial community is important. Components in human milk support microbial colonization. Further studies showing the preventative effect of breastfeeding for allergies are eagerly awaited. These will be important to unravel the components and mechanisms of human milk in allergy prevention and tolerance induction. Therefore, future research will have to focus on these protective compounds and mechanisms to support infants at risk for allergy development, to enable them to grow up free from allergies.

## Abbreviations

AMP: antimicrobial peptide; APC: antigen presenting cell; CRAMP: cathelicidin-related antimicrobial peptide; CCR: C–C chemokine receptor; CXCR1: CXC motif chemokine receptor 1; DC: dendritic cell; EPEC: enteropathogenic *Escherichia coli*; Foxp3+: forkhead box protein P3; IEC: intraepithelial cell; IL: interleukin; iTreg: induced Foxp3+ Treg cell; GALT: gut associated lymphoid tissue; LP: lamina propria; LPS: lipopolysaccharide; M cells: microfold cells; mLN: mesenteric lymph node; nTreg: natural Treg cell; OVA: ovalbumin; PP: Peyer's patch; SCFA: short-chain fatty acids; TGF-β: transforming growth factor-beta; TLR: Toll-like receptor; Treg: regulatory T cell.

## Funding

This review article was funded by HiPP GmbH & Co. Vertrieb KG, Pfaffenhofen, Germany.

## Availability of data and materials

This is a review article.

## Data availability statement

The data that support the findings of this study are freely available in Pubmed at https://pubmed.ncbi.nlm.nih.gov/, in the University of Nebraska Food Allergy Research and Resource Program, available at https://farrp.unl.edu/, and in the US National Library of Medicine Clinical Trials register, available at https://clinicaltrials.gov/.

## Author contribution

MH and OP initiated the concept. MS made the first draft. The other authors participated to the development of the document. All authors reviewed and approved the final manuscript.

## Ethics approval and consent to participate

Not applicable; the manuscript does not report on or involve the use of any animal or human data or tissue.

## Consent for publication

All authors agreed to publication of the work.

## Declaration of competing interest

With the exception of the financial support for workshop participation and manuscript preparation by HiPP GmbH & Co. Vertrieb KG, Pfaffenhofen, Germany, the authors report no conflicts of interest.
